# Laboratory evaluation of the effect of compaction method and compaction work on the performance of SMA-13 mixture

**DOI:** 10.1371/journal.pone.0265097

**Published:** 2022-03-08

**Authors:** Jinshun Xue, Yingjun Jiang, Yuanbiao Zheng, Shaohui Xiong

**Affiliations:** 1 Hubei Key Laboratory of Power System Design and Test for Electrical Vehicle, Hubei University of Arts and Science, Xiangyang, Hubei, China; 2 School of Civil Engineering and Architecture, Hubei University of Arts and Science, Xiangyang, Hubei, China; 3 Key Laboratory for Special Area Highway Engineering of Ministry of Education, Chang’an University, Xi’an, Shaanxi, China; 4 Ningbo Communications Planning Institute Co., Ltd., Ningbo, Zhejiang, China; Texas A&M University System, QATAR

## Abstract

The influence of compaction methods such as the Marshall compaction method (MCM), vibration compaction method (VCM) and gyration compaction method (GCM), on the performance of stone mastic asphalt (SMA-13) mixture has yet to be explored. Therefore, to compare the influences of compaction methods and work on the physical and mechanical properties of SMA-13 mixture, the volume parameters, mechanical properties, and gradation changes of SMA-13 mixture specimens prepared under different vibration compaction times, Marshall double-compaction numbers, and gyration compaction numbers were studied. The compaction method for SMA-13 mixture design was also proposed under the principle of optimum properties. Results demonstrate that the asphalt aggregate ratio and compaction work directly affect the volumetric properties (VV, VFA, and VMA) of asphalt mixture specimens while the raw material and mineral aggregate gradation were fixed. The influence of compaction work on physical properties is greater than that of asphalt aggregate ratio. The mechanical strength of VCM and GCM specimens is higher than that of MCM specimens under the same compaction work and the optimum asphalt aggregate ratio. With the increase in compaction work, the mechanical properties of SMA-13 mixture are improved at the same compaction method and the optimum asphalt aggregate ratio. The aggregate gradation of the SMA-13 mixture before and after compacted using VCM and GCM changes minimally compared with that of the SMA-13 mixture compacted by MCM. Thus, the compaction methods of VCM65 and GCM130 were recommended for SMA-13 mixture design.

## 1 Introduction

With the aggravating trend of China’s traffic toward the direction of “large flow, large-scale vehicles, heavy load, and overload” [1] and the frequent occurrence of extreme weather, early diseases of stone mastic asphalt (SMA) mixture pavement, such as rutting, cracks, and water damage, are becoming increasingly serious [2–4]. One reason for such a phenomenon is that the standard of 50 times Marshall double-compaction number is adopted for the mix proportion design of SMA mixture, which is evidently lagging behind the current traffic situation to prevent aggregate breakage due to excessive compaction number [5].

However, the Marshall compaction method (MCM), which is easy to operate for researchers, is mostly used in the research of SMA mixture at present [6]. Wu explored the effect of fiber types on the performance of SMA mixture under different ageing states [7]. The performance of SMA mixtures with different high-viscosity modified asphalts based on laboratory tests was evaluated by Luo [8]. SMA containing ceramic waste aggregate for cooling asphalt pavement was studied by Huang [9]. Nevertheless, previous studies have shown that the correlation between the engineering characteristics of Marshall specimens and field core specimens is less than 70% [10, 11]. Thus, the Hveem compaction methods, gyratory testing machine methods, and Superpave design methods are adopted for the compaction of asphalt mixture throughout the world [12, 13]. The compactability and water sensitivity of rubberized SMA mixtures with chemical warm mixture asphalt additive were evaluated using the Superpave gyratory compactor method (GCM) [14, 15]. The GCM for SMA mixture, specifically its design applicability and feasibility, was analyzed [16]. The mechanical differences of various asphalt mixtures were compared using GCM and MCM [15]. Although the specimens compacted using the above compaction methods show enhanced correlation with field samples, the equipment used for these compaction methods is considerably expensive for worldwide application [17].

Researchers have suggested asphalt mixture compaction via the vibration compaction method (VCM) to investigate the performance of mixtures. VCM can make asphalt mixtures have a skeleton dense structure, and the Marshall stability (MS) and crack resistance of asphalt mixtures can be significantly improved [18]. VCM not only makes the aggregate difficult to break but can also obtain compressive strength (*R*c) and splitting strength (*R*_T_) similar to those obtained using GCM [19]. Compared with the asphalt treated base mixture designed using MCM, the density, dynamic stability, shear strength (*σ*), tensile strength, and fatigue resistance of the asphalt mixture designed using the vertical vibration method are significantly increased [20]. However, the influence of compaction methods and work on the performance of SMA-13 mixture based on VCM, GCM, and MCM has yet to be explored. Hence, the physical properties, mechanical properties, and gradation changes of SMA-13 mixture specimens prepared under different vibration compaction times, Marshall double-compaction numbers, and gyration compaction numbers are studied. And the compaction method for SMA-13 mixture design was proposed under the principle of optimum properties.

## 2 Materials and experimental test

### 2.1 Materials

#### 2.1.1 Asphalt binders

SBS-modified asphalt supplied by a local manufacturer was used in this study, and its technical properties were tested in accordance with the requirements in JTG E20-2011 [21]. The test results are provided in Table 1.

**Table 1:** Technical properties of the SBS-modified asphalt.

Test item	Penetration,0.1mm	Penetration index	Softening point, °C	Ductility, cm
**Test results**	71	0.47	82	42.5
**Requirement**	60 ~ 80	≥-0.4	≥55	≥30
**Test standards**	T 0604–2011	T 0604–2011	T 0606–2011	T 0605–2011

#### 2.1.2 Aggregates

Granite aggregate stones from Shangluo City, Shaanxi Province, China were used as coarse aggregates (2.36–16 mm), and limestone aggregate stones from Shangluo City were used as fine aggregates and mineral powder. The technical properties were tested in accordance with the requirements in JTG E42-2005 [22], as presented in Tables 2–4.

**Table 2:** Technical properties of coarse aggregate.

Test items	Aggregate impact Value, %	LA Abrasion Value, %	Polishing value	Soundness, %
**Test results**	14.1	15.3	43	5.2
**Requirement**	≤26	≤28	≥40	≤12
**Test standards**	T 0316–2005	T 0317–2005	T 0321–2005	T 0314–2000

**Table 3:** Technical properties of fine aggregate.

Test items	Sand equivalent, %	Angularity, s	Methylene blue value, g/kg
**Test results**	89.5	32.8	2.0
**Requirement**	≥60	≥30	≤2.5
**Test standards**	T 0334–2005	T 0345–2005	T 0349–2005

**Table 4:** Technical properties of mineral power.

Test items	Bulk Specific Gravity, g/cm^3^	Plasticity index	Hydrophilic con cent
**Test results**	2.706	0.58	3.0
**Requirement**	≥2.5	≤1.0	≤4
**Test standards**	T 0352–2000	T 0354–2000	T 0353–2000

#### 2.1.3 Fiber

Lignin fiber supplied by a local manufacturer from Jiangsu Province, China was used in this study, in which the technical properties were tested in accordance with the requirements in JTG F40-2004 [23]. The test results are provided in Table 5. The fiber content of the SMA-13 mixture was 0.3% in accordance with relevant experiences.

**Table 5:** Technical properties of lignin fiber.

Test items	Average Length, mm	Fiber Diameter, mm	Elastic Modulus, Gpa	Elongation at Break, %
**Test results**	1.0	0.045	3.5	20
**Test standards**	JT/T 776.1	GB/T 7690.5	GB/T 20310	GB/T 20310

### 2.2 Mixture gradation

The gradation of SMA-13 mixture listed in Table 6 was applied in this study.

**Table 6:** Mineral aggregate gradation of SMA-13 asphalt mixture.

**Sieve size, mm**	16	13.2	9.5	4.75	2.36	1.18	0.6	0.3	0.15	0.075
**Passing rates, %**	100	91.2	63.5	28.8	24.5	20.4	15.2	13.3	11.6	9.5

### 2.3 Experimental program

The asphalt aggregate ratio commonly used for SMA-13 mixture is 5.0%– 6.5%, such that SMA-13 mixture specimens with five asphalt aggregate ratios, 5.1%, 5.4%, 5.7%, 6.0%, and 6.3%, were produced using MCM, GCM, and VCM. Different compaction numbers were used for the specimens compacted using MCM and GCM, and different beat times were used for the specimen compacted using VCM.

**Fig 1:**
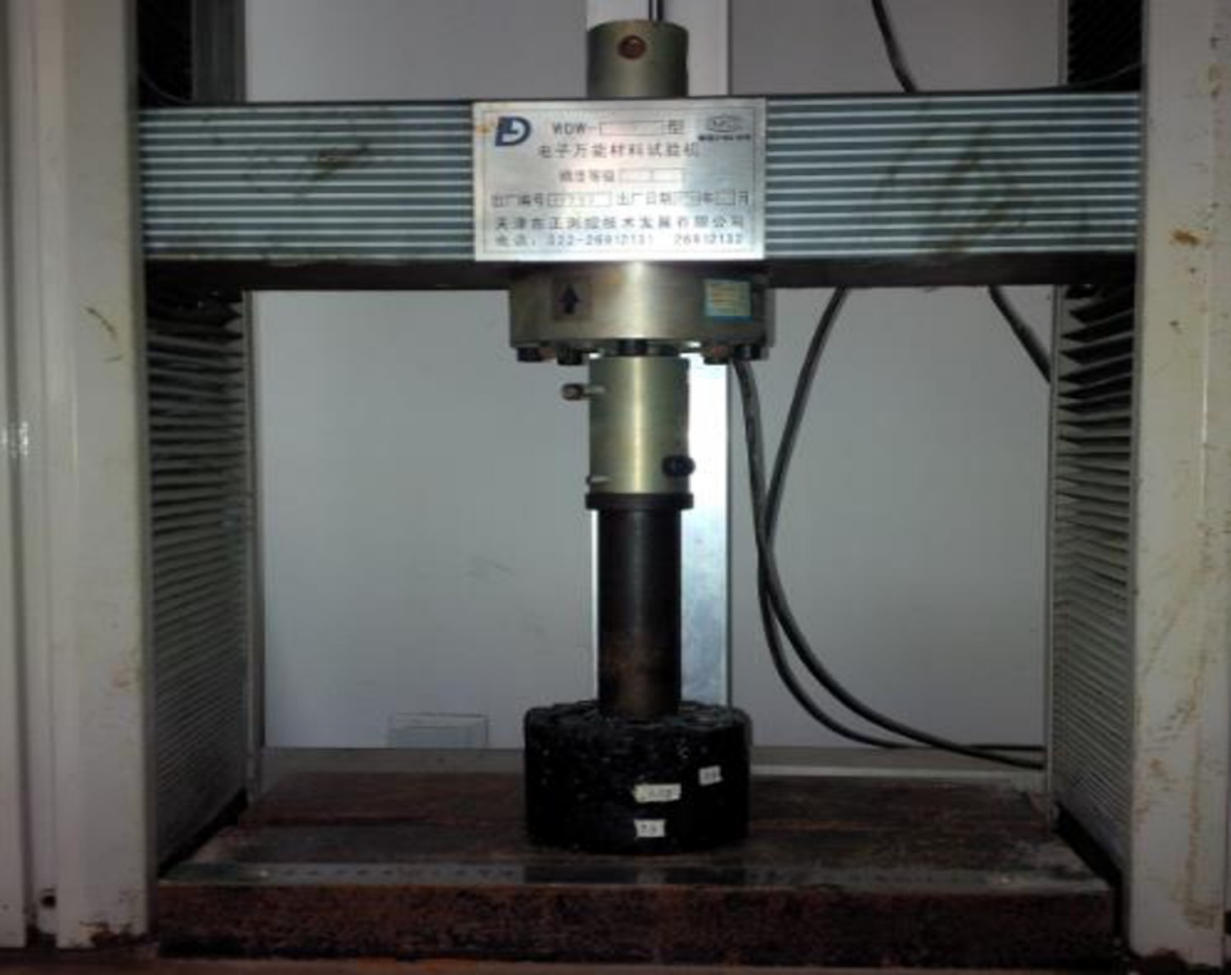
The uniaxial penetration test system.

The effects of compaction method and the compaction effect on volume parameters (such as density, volume of air voids (VV), and voids filled with asphalt (VFA), voids in mineral aggregate (VMA)) and mechanical properties (such as 60°C Marshall stability, 25°C compressive strength, -10°C splitting strength and 60°C shear strength) were evaluated. Shear strength was measured via the uniaxial penetration test, and other technical indexes were measured in accordance with the testing methods of the Ministry of Transport of the People’s Republic of China for bitumen and bituminous mixtures for highway engineering (Table 7) [21]. The uniaxial penetration test is illustrated in Fig 1. The electronic universal testing machine has a 38 mm diameter of the indenter and 1 mm/min loading rate. The cylindrical specimen used for the uniaxial penetration test was placed in an oven with an air temperature of 60°C for 4 hours to 6 hours. The shear strength was calculated in accordance with Formula (1). The performance tests were performed four times, and the results were examined and averaged through Grubbs’ method.

**Table 7:** Test standards of technical indexes.

Technical indexes	Test standards	Technical indexes	Test standards
Density	T 0706–2011	Compressive strength	T 0713–2000
Marshall stability	T 0709–2011	Splitting strength	T 0716–2011


$$\tau_{d}=1.356P/(\pi \cdot \Phi^{2})$$ (1)

where *τ*_d_ is the shear strength (MPa), *P* is the maximum stress at failure of the specimen (N), and *Φ* is the diameter of specimen (mm).

### 2.4 Specimen preparation methods

This study adopted the MCM established in JTGE20-2011. The specimens were prepared using a Marshall compactor, in which the working parameters are listed in Table 8. MCM as an uncomplicated and inexpensive compaction method, widely adopted for SMA mixtures worldwide, but the Marshall compactor hammer drop impact results in the breakage of aggregate during the compaction process; consequently, the actual pavement compaction situation cannot be effectively simulated [13]. On the contrary, GCM could better efficiently simulate the stress state of a pavement surface under the action of loadings. Specimens compacted by using GCM had a good correlation with the field core samples from the pavement. The compaction of specimens by using SGC met the requirements in JTGE20-2011. The parameters of the gyratory compactor are listed in Table 9. The instrument adopted in VCM was vertical vibratory testing equipment (VVTE), which applied only vertical force during the compaction procedure. The working parameters of the VVTE are listed in Table 10.

**Table 8:** Working parameters of the Marshall compactor.

Compaction hammer, g	Drop height of hammer, cm	Working frequency, times•min^-1^	Specimen size, mm
4536	47.5	60	*Φ*101.6×*h*63.5

**Table 9:** Working parameters of the gyratory compactor.

Vertical pressure, kPa	Compaction rotation angle,°	Rotation speed, r•min^-1^	Specimen size, mm
600	1.25	30	*Φ*100×*h*63.5

**Table 10:** Working parameters of the VVTE.

Working frequency, Hz	Upper equipment weight, Kg	Nether equipment weight, Kg	Static eccentricity, Kg•m	Specimen size, mm
37	167	108	0.109	*Φ*100×*h*63.5

## 3 Relationship between compaction number or time and the density of SMA-13 mixture compacted using different compaction methods

**Fig 2:**
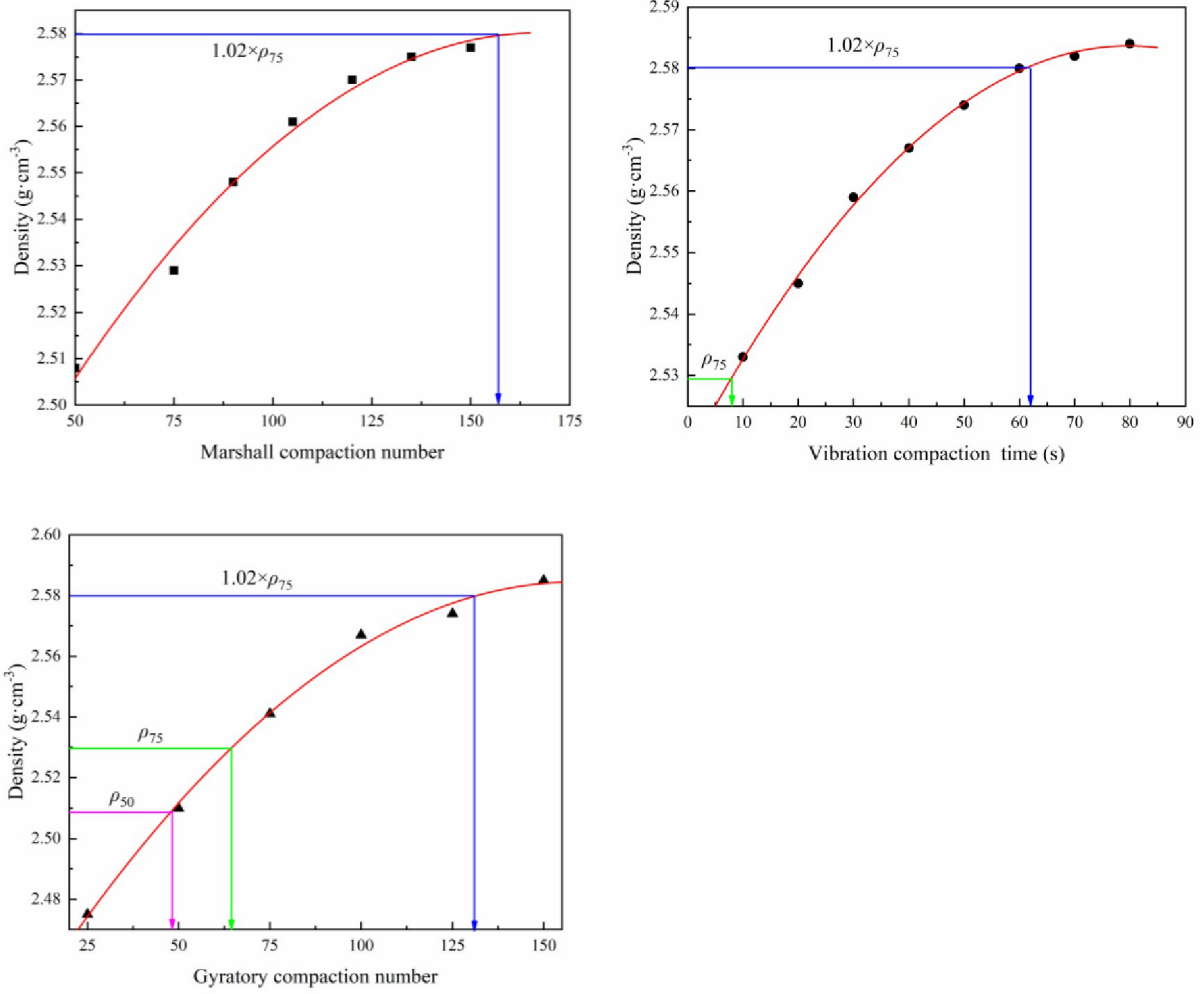
The relationship between compaction number (time) and specimen density. (a) Density versus Marshall compaction number (b) Density versus vibration compaction time. (c) Density versus gyratory compaction number.

The compaction numbers of 50, 75, 90, 105, 120, 135, and 150 were used for the specimens compacted using MCM; the compaction times of 10 s, 20 s, 30 s, 40 s, 50 s, 60 s, 70 s, and 80 s were used for the specimens compacted using VCM; the gyratory compaction numbers of 25, 50, 75, 100, 125, and 150 were used for the specimens compacted using GCM. The gross bulk density of the specimens was measured. The relationship between compaction number or time and the density of the specimens is shown in Fig 2.

From Fig 2(A), the Marshall double-compaction number corresponding to the density in the heavy traffic compaction standard is 155, which can be interpreted as that double-compaction number 155 can reach the density specified in the heavy traffic compaction standard [10]. Accordingly, the compaction method with Marshall double-compaction number 155 is called heavy MCM, and the specimens compacted using this method are called heavy MCM specimens. Fig 2(B) shows that when the asphalt stone ratio is 5.7%, the vibration compaction time corresponding to the density of Marshall double-compaction number 75 is 8s, and the vibration compaction time corresponding to the density in the heavy traffic compaction standard is 63s. Hence, the vibration compaction times corresponding to the density of Marshall double-compaction number 75 and heavy traffic compaction standard are 10s and 65s, respectively. Fig 2(C) presents that the gyratory compaction numbers corresponding to the densities of Marshall double-compaction numbers 50, 75, and 155 are 50, 65, and 130, respectively.

For simplicity, the specimens compacted using a Marshall double-compaction number of 75, vibration compaction time of 65s, and gyratory compaction number of 65 were replaced with MCM75, VCM65, and GCM 65, respectively. The same replacement applied to the others.

## 4 Effect of compaction on the physical characteristics of SMA-13 mixture

The compaction method and work have a considerable influence on the particle arrangement of asphalt mixtures and the breakage of aggregates in the compaction process, which can inevitably affect the volume parameters of the mixtures. Thus, the structure of mixture specimens and the mechanical properties and optimal asphalt aggregate ratio of mixtures are also affected. The effects of compaction method and work on the physical properties of asphalt mixtures are studied under certain material composition.

### 4.1 Effect of compaction on the density of SMA-13 mixture

**Fig 3:**
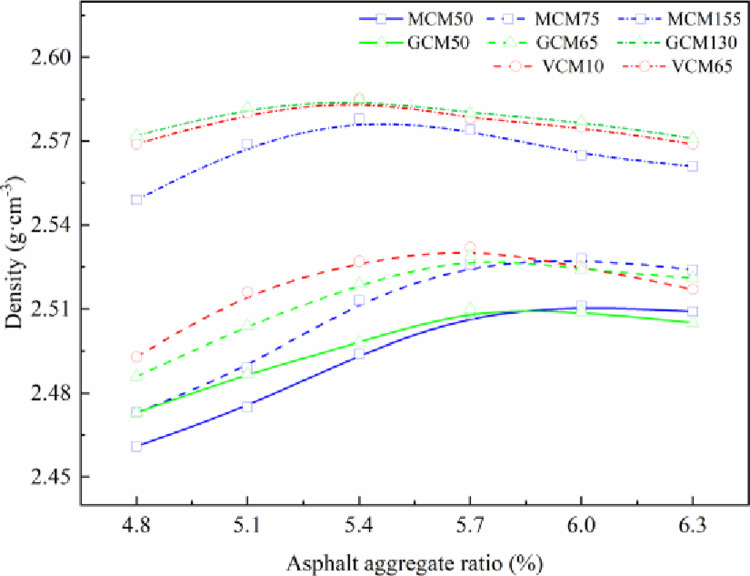
Relationship curve between specimen density and asphalt aggregate ratio.

The variation in density of SMA-13 mixture with asphalt aggregate ratio under different compaction methods and compaction work is shown in Fig 3.

Fig 3 demonstrates that the density of asphalt mixture specimens is a function of asphalt aggregate ratio and compaction work while the raw material and mineral aggregate gradation are fixed. A parabola relationship exists between the density of SMA-13 mixture and the asphalt aggregate ratio for specimens under different compaction methods. When the asphalt stone ratio of the mixture is small, the asphalt film thickness of the asphalt mixture is small, the friction among aggregate particles is large, and the aggregate cannot move fully. Consequently, the density of the SMA-13 mixture specimen is low. With the increase in the asphalt aggregate ratio of the mixture, the thickness of the asphalt film increases. Under the action of external compaction work, the lubrication effect intensifies gradually, and the aggregate particles in the mixture move fully. As a result, the density of SMA-13 mixture specimens increases gradually. After the density of a specimen reaches the peak, if the asphalt content continues to increase, the asphalt will hinder the proximity of aggregate particles and offset part of the compaction work. Therefore, when the asphalt aggregate ratio is exceedingly large, the density of the mixture specimen will decrease [24].

Although the compaction mechanisms of GCM50 and MCM50 are not identical, the variation in the density of SMA-13 mixture specimens compacted using the two methods with asphalt aggregate ratio is basically the same. The asphalt aggregate ratio of the maximum density of specimens is 5.8%, that of the maximum density of VCM10, GCM65, and MCM75 specimens is 5.7%, and that of the maximum density of VCM65, GCM130, and MCM155 specimens is 5.4%. This result occurs because although the compaction methods are different, the compaction work of diverse compaction methods is basically the same. Nonetheless, the compaction work of VCM65, GCM130, and MCM155 is greater than that of VCM10, GCM65, and MCM75, and the maximum density of asphalt mixtures increases with the increase in compaction work. Specifically, the average density of VCM65, GCM130, and MCM155 specimens is 1.02 times that of VCM10, GCM65, and MCM75 specimens; the maximum density corresponding to the asphalt aggregate ratio decreases with the increase in compaction work. The asphalt aggregate ratio of the maximum density of VCM10, GCM65, and MCM75 specimens is 5.7%, and the asphalt aggregate ratio of the maximum density of VCM65, GCM130, and MCM155 specimens is 5.4%. This result shows that when the compaction work is small, the aggregates hardly reach the close contact state, and substantial asphalt lubrication and filling are needed to increase the density of asphalt mixtures.

### 4.2 Effect of compaction on the VV of SMA-13 mixture

**Fig 4:**
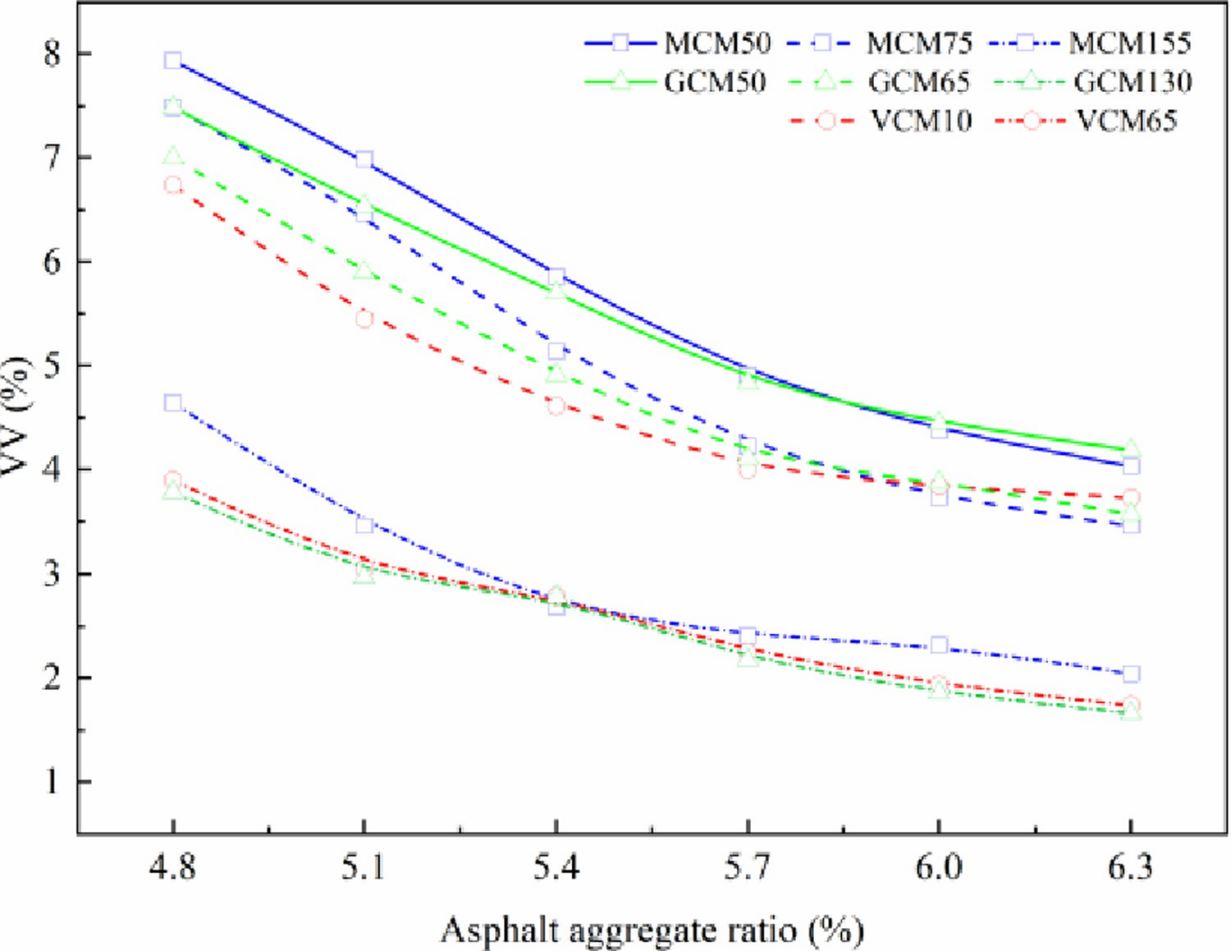
The relationship curve between VV and asphalt aggregate ratio.

As an important volume parameter index of asphalt mixtures, VV has been considered to have direct effects, such as water seepage and aging, on the durability of pavement. VV becomes a weak point in pavements; the voids are prone to stress concentration and microdamage under the load action. The repeated action of stress gradually accumulates and expands the microdamage, which continuously reduces the effective stress bearing area and causes further damage after repeated action for a certain number of times. The variation in VV with asphalt aggregate ratio under different compaction methods and compaction work is shown in Fig 4.

Fig 4 indicates the result of VV that decreases linearly as the asphalt aggregate ratio increases under the same compaction methods. Although the compaction mechanism differs, the specimens’ VV variation is mostly the same over the asphalt aggregate ratio under different compaction methods, and the specimens’ VV decreases with the increase in compaction work. With the increase in asphalt aggregate ratio, the voids of the mixture are filled with asphalt, resulting decreased VV. Compared with the VV (1.4%–3.8%) of VCM65, GCM130, and MCM155, the VV (2.7%–6.7%) of VCM10, GCM65, and MCM75 decreases by 2.1%, and the VV (3.3%–7.1%) of GCM50 and MCM50 decreases by 2.8% under the same asphalt aggregate ratio. With the increase in compaction work, the skeleton structure of the asphalt mixture is denser, resulting in decreased VV of the asphalt mixture.

### 4.3 Effect of compaction on the VMA of SMA-13 mixture

**Fig 5:**
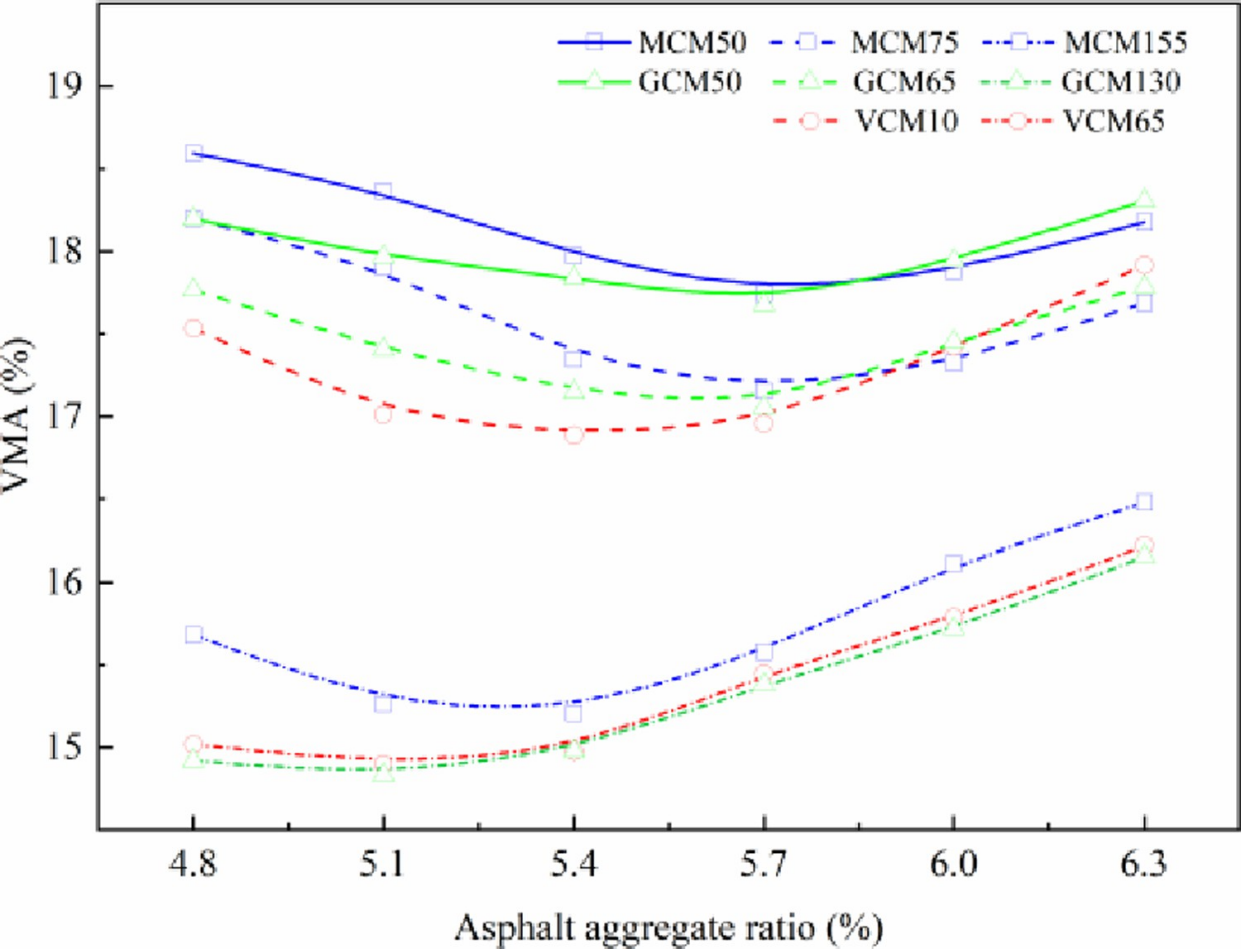
The relationship curve between VMA and asphalt aggregate ratio.

VMA is a key index in the design process of SMA-13 mixture, which is directly related to asphalt aggregate ratio and is one of the most important indexes reflecting the overall quality of the asphalt mixture. The variation in VMA with asphalt aggregate ratio under different compaction methods and compaction work is shown in Fig 5.

Fig 5 illustrates that the asphalt aggregate ratio and compaction work have direct influences on the VMA of the asphalt mixture while the raw material and mineral aggregate gradation are fixed. The VMA of SMA-13 mixture changes in a concave curve with the increase in asphalt aggregate ratio under different compaction methods. Before the VMA of the asphalt mixture reaches the minimum value, with the increase in asphalt content in the mixture, the asphalt lubrication increases, the friction among mineral aggregate particles decreases, the particles of the asphalt mixture are easy to displace and rearrange under external compaction, and the VMA of the asphalt mixture decreases. When VMA reaches the minimum value, the asphalt content continues to increase, which hinders the convergence of mineral aggregate particles, such that the VMA of the asphalt mixture increases. Although the compaction mechanism differs, the specimens’ VMA variation is much the same over the asphalt aggregate ratios under different compaction methods. The asphalt aggregate ratio corresponding to the minimum VMA of SMA-13 mixture decreases with the increase in compaction work. The peak value of the minimum VMA of MS75 and MS155 appears near the asphalt aggregate ratios of 5.7% and 5.3%, respectively; the peak value of the minimum VMA of the MS50 specimen appears at the asphalt aggregate ratio of 5.8%. The specimens’ VMA decreases with the increase in compaction work. Compared with the VMA (14.4%–16.1%) of VCM65, GCM130, and MCM155, the VMA (16.3%–17.6%) of VCM10, GCM65, and MCM75 is decreases 1.7%, and the VMA (17.2%–18.1%) of GCM50 and MCM50 is decreases 2.8% under the same asphalt aggregate ratio.

### 4.4 Effect of compaction on the VFA of SMA-13 mixture

**Fig 6:**
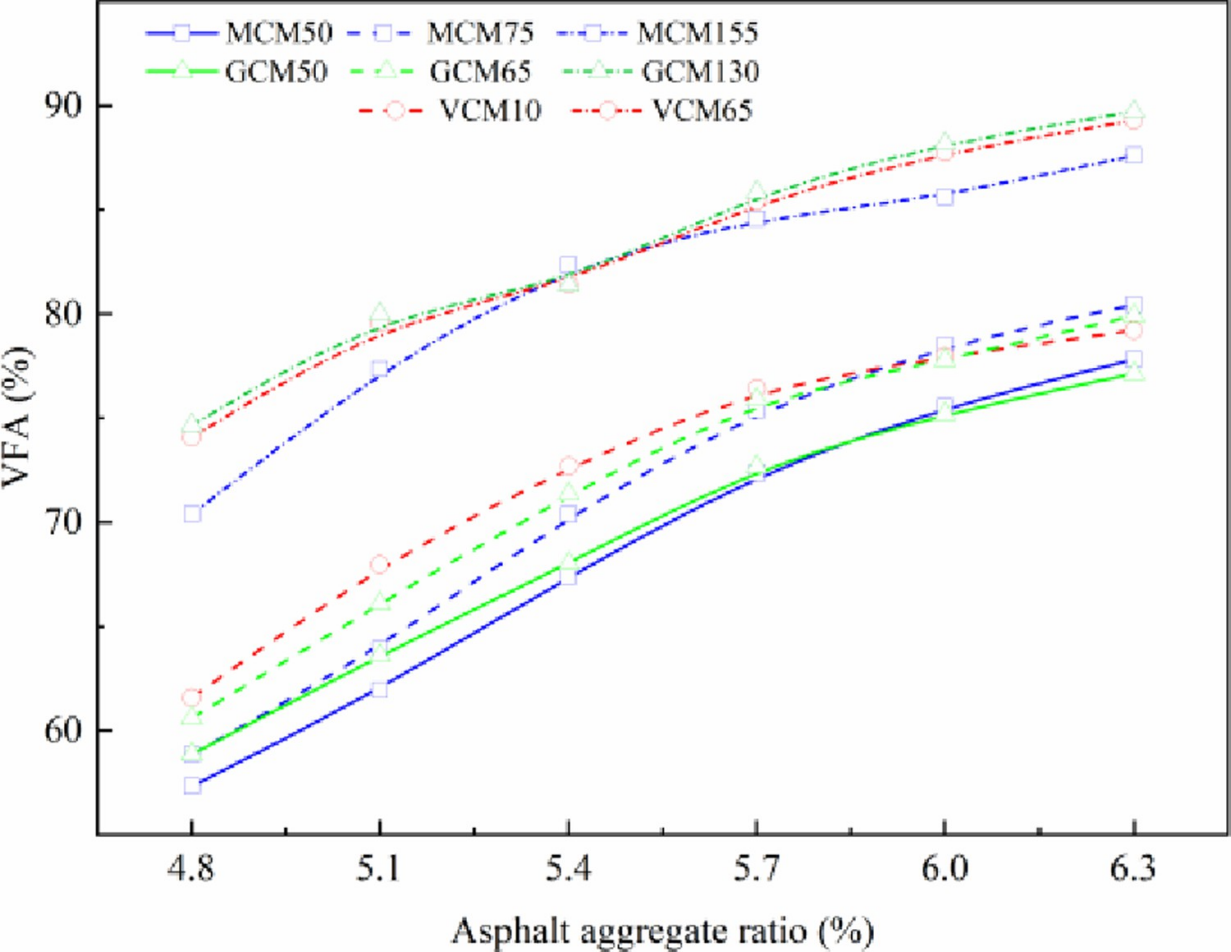
The relationship curve between VFA and asphalt aggregate ratio.

Asphalt can be divided into structural and free asphalt. Structural asphalt has greater cohesion than free asphalt, and free asphalt can fill voids and reduce pavement water seepage and aging. Excessive free asphalt will affect the pavement strength; insufficient free asphalt will affect the pavement durability [25]. Therefore, VFA has a significant impact on the performance of asphalt mixtures. The variation in VFA with asphalt aggregate ratio under different compaction methods and compaction work is shown in Fig 6.

Fig 6 depicts that, on either compaction method, with the increase in asphalt aggregate ratio, the asphalt gradually fills the mineral aggregate gap of the specimen, and the VFA of the specimen increases. Although the compaction mechanism differs, the specimens’ VFA variation is much the same over the asphalt aggregate ratios under different compaction methods. The VFA of the specimens increases with the increase in compaction work under the same asphalt aggregate ratio. Compared with the VFA (74.7%–91.0%) of VCM65, GCM130, and MCM155, the VFA (62.1%–84.4%) of VCM10, GCM65, and MCM75 is improved by 9.6%, and the VFA (60.4%–81.5%) of GCM50 and MCM50 is improved by 11.9% under the same asphalt aggregate ratio.

In conclusion, the volumetric properties of asphalt mixture specimens are a function of asphalt aggregate ratio and compaction work while the raw material and mineral aggregate gradation are fixed. When the asphalt content is the same but the compaction work is different, the volumetric properties (VV, VMA, and VFA) of the compacted mixture are also different. Different compaction standards should have distinct volumetric properties and the optimum asphalt aggregate ratio.

## 5 Effect of compaction on the mechanical properties of SMA-13 mixture

### 5.1 Effect of compaction methods on the mechanical properties of SMA-13 mixture

**Fig 7:**
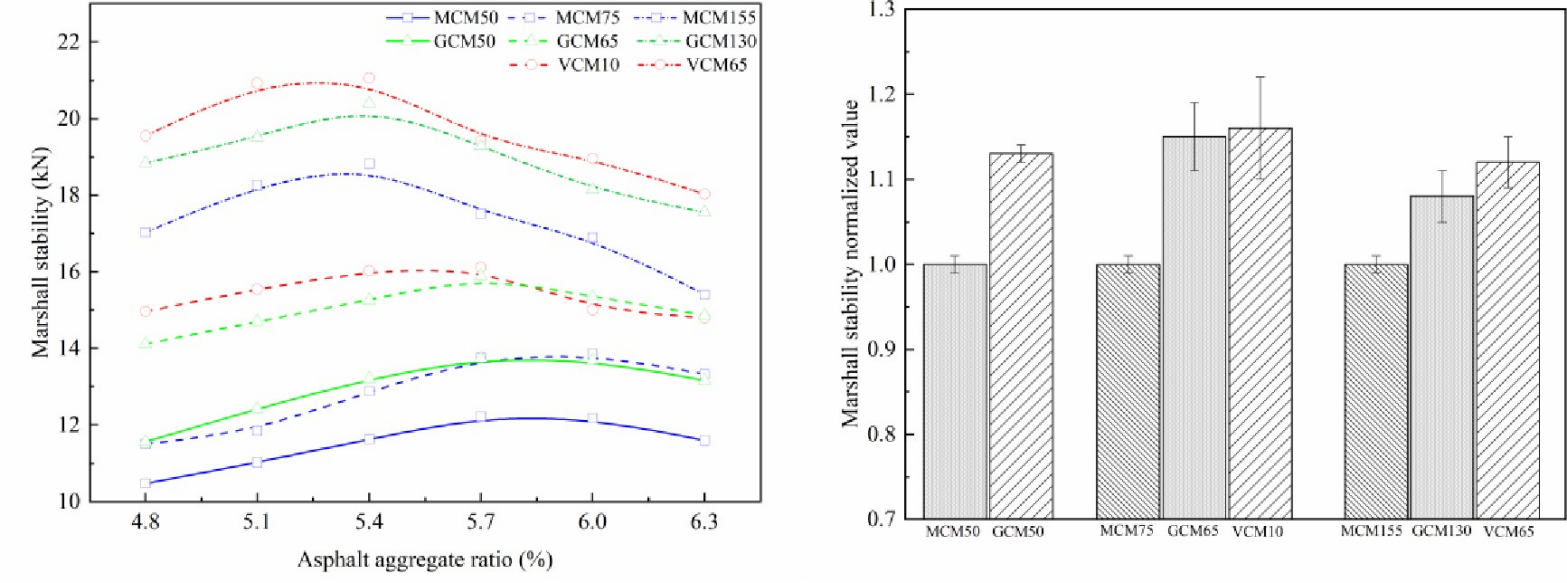
Effect of compaction on Marshall stability under different compaction methods. (a) Marshall stability versus asphalt aggregate ratios. (b) Marshall stability normalized value.

**Fig 8:**
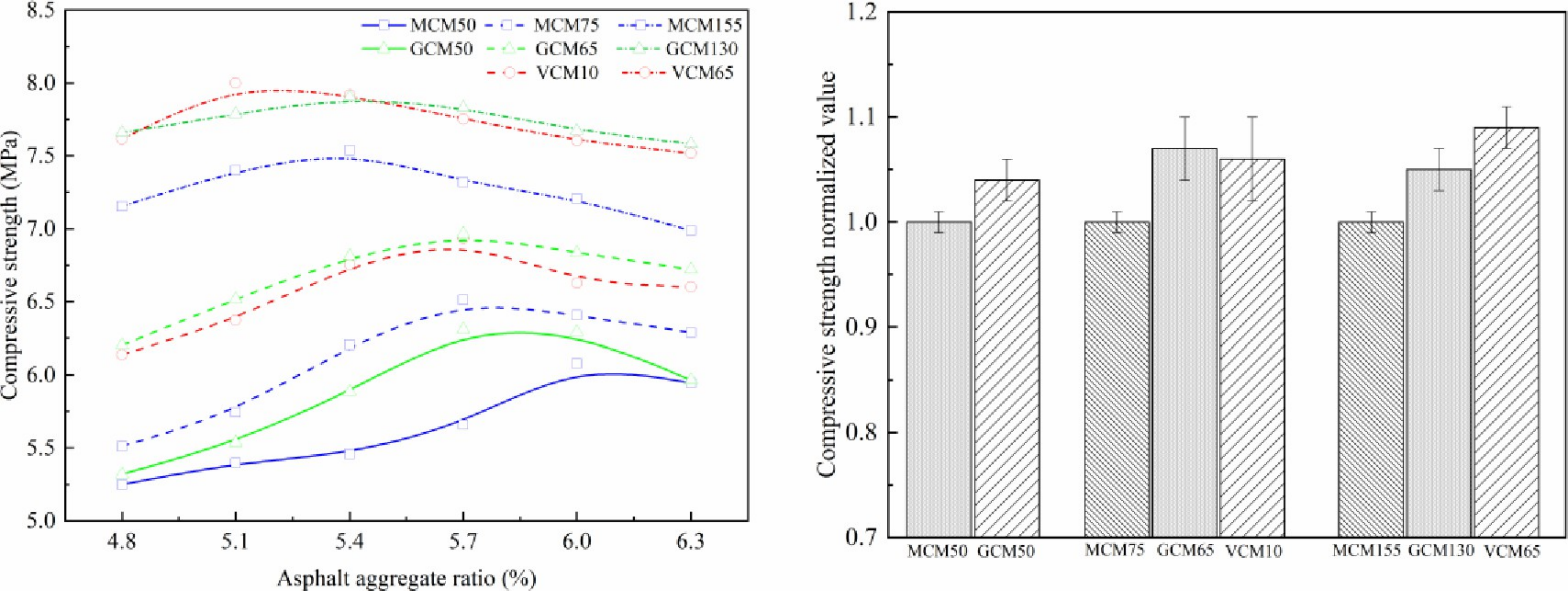
Effect of compaction on compressive strength under different compaction methods. (a)Compressive strength versus asphalt-aggregate ratios (b) Compressive strength normalized value.

**Fig 9:**
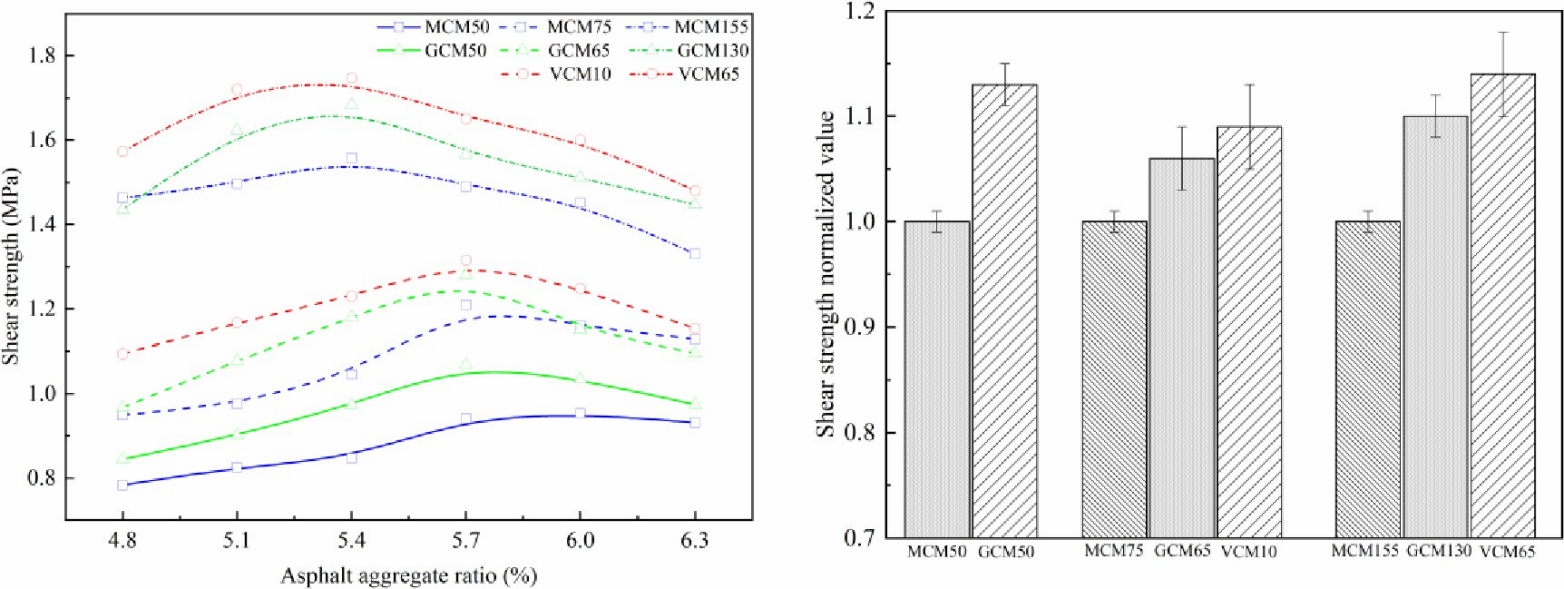
Effect of compaction on shear strength under different compaction methods. (a) Shear strength versus asphalt-aggregate ratios (b) Shear strength normalized value.

**Fig 10:**
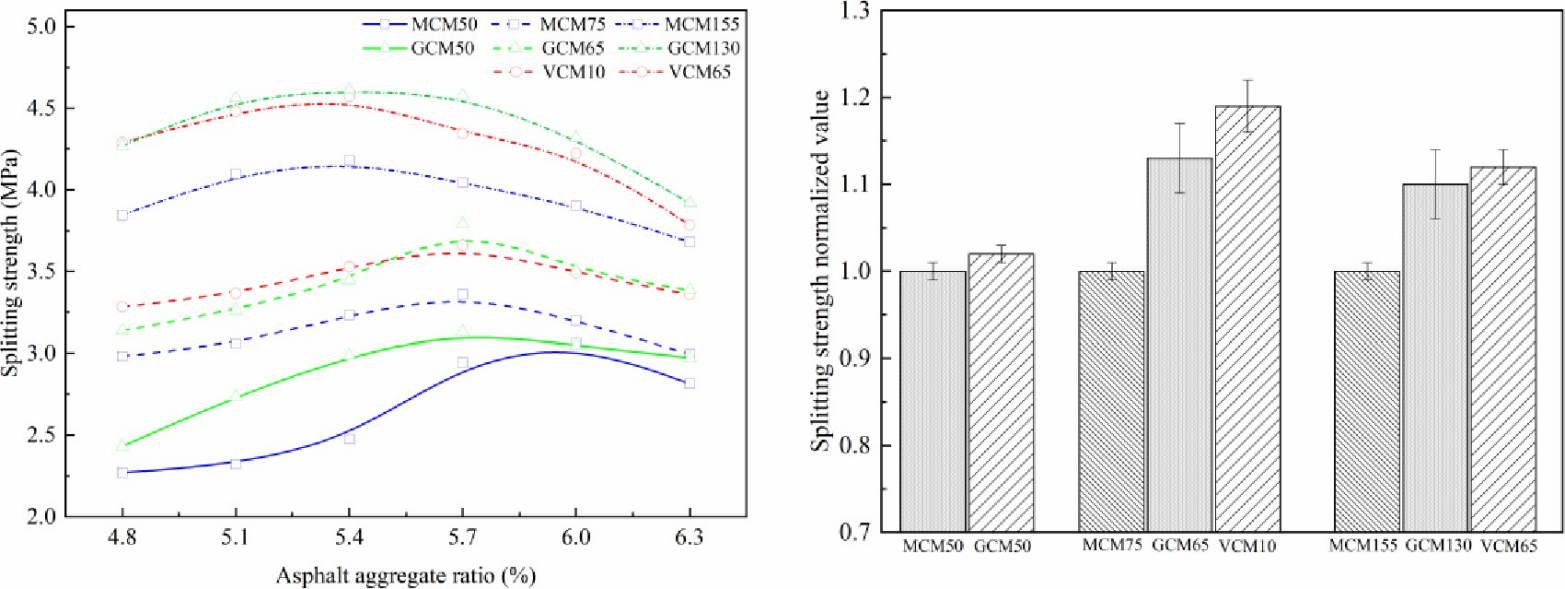
Effect of compaction on splitting strength under different compaction methods. (a) Splitting strength versus asphalt-aggregate ratios. (b) Splitting strength normalized value.

The effect of compaction on mechanical properties under different compaction methods is shown in Figs 7–10. The relationship curve between mechanical properties and asphalt aggregate ratio under different compaction methods is presented in Fig 7(A), and the normalized values of mechanical property peak of different compaction methods are shown in Fig 7(B).

Figs 7–10 depict that the mechanical properties of SMA-13 mixture change in a convex curve with the increase in asphalt aggregate ratio under the same compaction method. The mechanical properties of the asphalt mixture specimens compacted using VCM and GCM are higher than that of the asphalt mixture specimen compacted using MCM.

When the asphalt aggregate ratio of SMA-13 mixture is small, its VMA and VV are large, the VFA is low, and the mechanical properties are low. With the increase in asphalt aggregate ratio, the thickness of the asphalt film increases gradually, the friction among aggregate particles decreases, the VMA and VV of the mixture decrease, the VFA increases, and the mechanical properties first increase and then decline as a single-peak curve. The content of free asphalt increases and the cohesion of the asphalt weakens with the increase in asphalt aggregate ratio after the mechanical properties peak.

The asphalt aggregate ratio of the mechanical property peak of MCM50 and GCM50 specimens is 5.8%, that of VCM10, GCM65, and MCM75 specimens is 5.7%, and that of VCM65, GCM130, and MCM155 specimens is 5.4%. This result shows that the optimum asphalt aggregate ratio of the mechanical property peak is a function of compaction work. The greater the compaction work is, the smaller the optimum asphalt aggregate ratio of the SMA-13 mixture is. When the compaction work is small, the friction of aggregates is high, and substantial asphalt is needed to make the aggregate dense. Consequently, the optimum asphalt aggregate ratio corresponding to the optimum mechanical properties of SMA-13 mixture under various compaction methods is different. Different compaction methods have distinct mechanical properties under the optimum asphalt aggregate ratio and the same compaction work.

Under the same compaction work, the Marshall stability, compressive strength, shear strength and splitting strength of VCM10 specimens are 1.16, 1.06, 1.09, and 1.19 times higher than those of MCM75 specimens, respectively, with an average value of 1.13 times. The Marshall stability, compressive strength, shear strength and splitting strength of GCM65 specimens are 1.15, 1.07, 1.06, and 1.13 times higher than those of MCM75 specimens, respectively, with an average value of 1.10 times. The mechanical properties of GCM50 specimens are 1.08 times higher than those of MCM50 specimens; the mechanical properties of GCM130 specimens are 1.08 times higher than those of MCM155 specimens; the mechanical properties of VCM65 specimens are 1.12 times higher than those of MCM155 specimens. This result shows that the compaction method plays an important role in the positioning and arrangement of internal particle structure, and VCM and GCM are more conducive to the rearrangement of internal particles and the formation of stable structure than MCM [26].

### 5.2 Effect of compaction work on the mechanical properties of SMA-13 mixture

**Fig 11:**
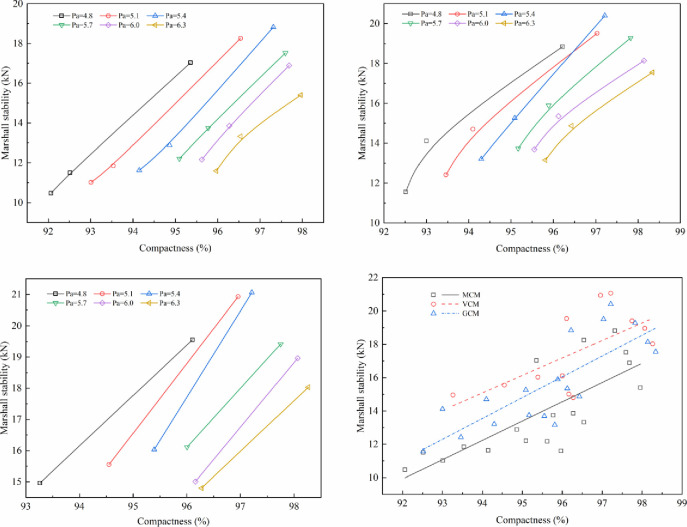
The relationship between Marshall stability and compactness. (a)Marshall stability versus compactness under MCM (b) Marshall stability versus compactness under GCM. (c)Marshall stability versus compactness under VCM (d) Relationship between Marshall stability and compactness.

**Fig 12:**
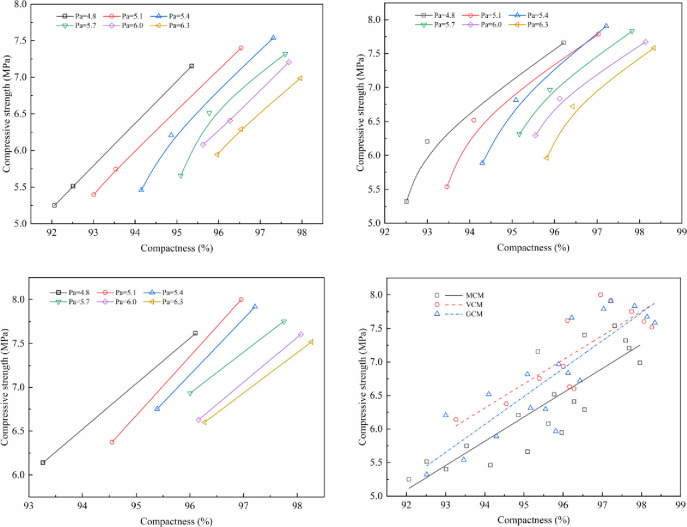
The relationship between compressive strength and compactness. (a) Compressive strength versus compactness under MCM (b) Compressive strength versus compactness under GCM. (c)Compressive strength versus compactness under VCM (d) Relationship between compressive strength and compactness.

**Fig 13:**
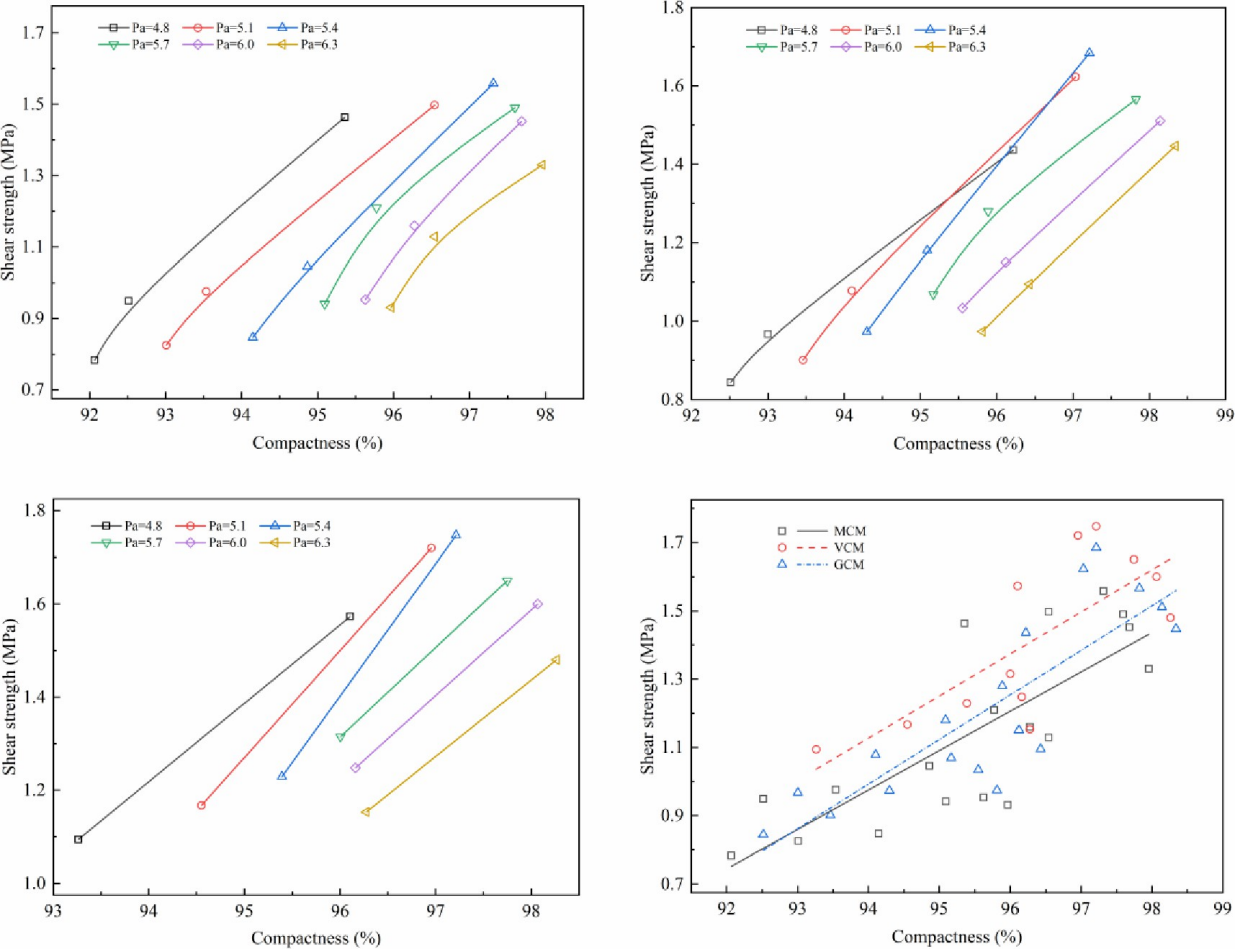
The relationship between shear strength and compactness. (a) Shear strength versus compactness under MCM (b) Shear strength versus compactness under GCM. (c)Shear strength versus compactness under VCM (d) Relationship between shear strength and compactness.

**Fig 14:**
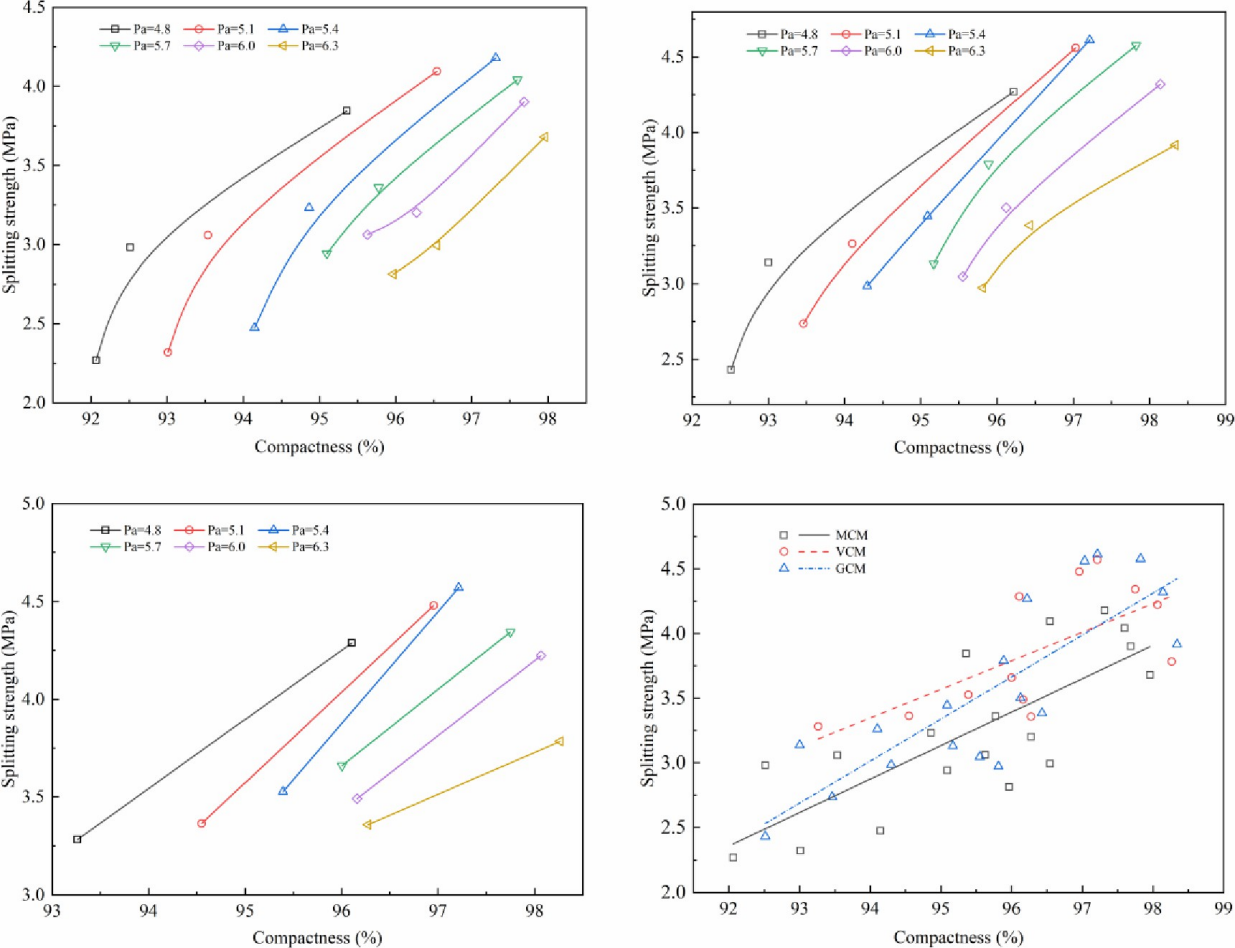
The relationship between splitting strength and compactness. (a) Splitting strength versus compactness under MCM (b) Splitting strength versus compactness under GCM. (c)Splitting strength versus compactness under VCM (d) Relationship between splitting strength and compactness.

The effect of compaction work on mechanical properties under different compaction methods is shown in Figs 11–14.

Figs 11–14 illustrate the relationship between the compactness and the mechanical properties of SMA-13 mixture with various asphalt aggregate ratios under different compaction methods. Comparison of specimens shows that the mechanical properties of SMA-13 mixture increase linearly as the compactness increases under the same compaction method and asphalt aggregate ratio. If the compactness of the MCM-compacted SMA-13 mixture increases by 1%, the Marshall stability, compressive strength, shear strength and splitting strength are improved on average by 11.3%, 8.0%, 18.0%, and 13.7%, respectively. If the compactness of the GCM-compacted SMA-13 mixture increases by 1%, the Marshall stability, compressive strength, shear strength and splitting strength are improved on average by 11.7%, 7.1%, 15.5%, and 11.0%, respectively. If the compactness of the VCM-compacted SMA-13 mixture is increases 1%, the Marshall stability, compressive strength, shear strength and splitting strength are improved on average by 8.1%, 6.5%, 14.0%, and 7.6%, respectively. These results show that at a certain asphalt aggregate ratio, the increased compactness of SMA-13 mixture leads to improved mechanical properties. Higher compactness means higher density of SMA-13 mixture, and the contact among aggregates of the mixture is denser. The specimens with stronger mechanical properties, whether at a low or high asphalt aggregate ratio, shows better performance.

At the same compactness of SMA-13 mixture, different compaction methods have diverse mechanical properties. The mechanical properties of the mixture compacted using VCM are best compared with those of the mixtures compacted using GCM and MCM. Therefore, the compaction method plays a key role in the positioning and arrangement of internal particle structure, and VCM and GCM are more conducive to the rearrangement of internal particles and the formation of stable structure than MCM.

## 6 Effect of compaction on the gradation of SMA-13 mixture

**Fig 15:**
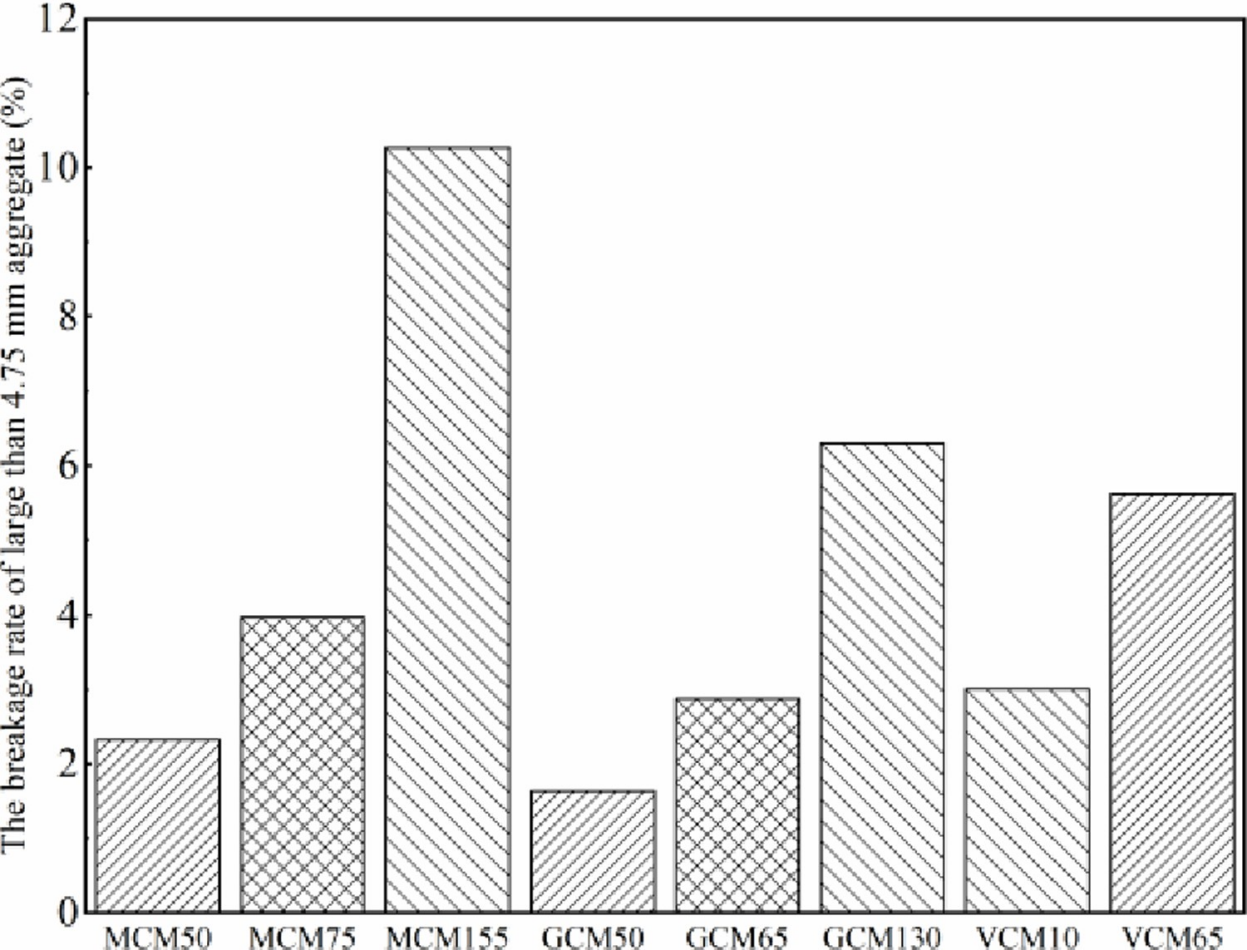
The breakage of large 4.75 mm aggregates of different compaction methods.

The performance of SMA-13 mixture, especially the rutting resistance of the mixture, is affected by gradation. The compaction method and work influence the gradation of the asphalt mixture during the compaction progress and hence the performance of the mixture. The gradation of SMA-13 mixture with different compaction methods and compaction work is shown in Table 11 and Fig 15.

**Table 11:** Effect of different compaction method and compaction work on the gradation of SMA-13 mixture.

**Compaction methods**	**Percentage passing (%) for sieve size (mm)**									
	16	13.2	9.5	4.75	2.36	1.18	0.6	0.3	0.15	0.075
**Original gradation**	100	91.2	63.5	28.8	24.5	20.4	15.2	13.3	11.6	9.5
**Gradation of MCM50**	100	92.4	65.0	30.0	25.0	20.1	16.5	13.8	11.3	9.5
**Gradation of MCM75**	100	92.8	65.7	31.3	26.1	20.9	16.7	13.6	11.0	9.5
**Gradation of MCM155**	100	94.2	67.9	34.8	27.1	21.0	17.3	14.5	11.8	9.7
**Gradation of VCM10**	100	92.2	64.7	30.6	24.8	19.7	16.6	13.6	11.6	9.5
**Gradation of VCM65**	100	92.7	66.7	33.9	26.5	20.3	16.5	14.4	11.9	9.6
**Gradation of GCM50**	100	95.4	63.5	28.2	21.3	19.7	16.6	13.6	12.6	10.5
**Gradation of GCM65**	100	95.5	63.8	29.1	21.6	19.8	16.7	13.6	12.6	10.4
**Gradation of GCM130**	100	96.4	66.2	31.6	22.6	20.5	17.3	14.1	13.1	10.9

Table 11 indicates that the gradations of SMA-13 mixtures compacted using different compaction methods are changed compared with the original gradation, which shows that the aggregates of the mixtures are crushed during the compaction progress. When the compaction method of SMA-13 mixture is the same, higher compaction work leads to greater breakage of the larger than 4.75 mm size aggregates of SMA-13 mixture specimens. If the compactness of the SMA-13 mixture increases by1%, the breakage of the specimens compacted using MCM, GCM, and VCM increases on average by 2.8%, 1.8%, and 1.2%, respectively.

From Fig 15, in the same compaction work, the aggregate gradation of the SMA-13 mixture before and after compacted using VCM and MCM changes minimally compared with that of the SMA-13 mixture compacted using MCM. In particular, the breakage of aggregates with a size larger than 4.75 mm is evidently smaller than that of the MCM specimen. Compared with the MCM specimen, the breakage of larger than 4.75 mm size aggregates of the GCM specimen is reduced by 1.9%, and that of the VGM specimen is reduced by 2.8%. Such a phenomenon can be analyzed as follows. The impact force is provided by the Marshall hammer during the MCM process, which drives the aggregate particles to move downward and to be embedded in the voids among the aggregates to achieve the density of SMA-13 mixture. Therefore, the breakage of coarse aggregates is evidently at both ends of the MCM specimen. Vibration pressure wave can be provided by VVTE during the VCM progress, then the asphalt mixture transits from the initial static state to the moving state under the action of vibration pressure wave. The friction among aggregate particles gradually changes from the initial static friction state to the dynamic friction state. The internal friction resistance decreases, *σ* declines, the compressive resistance diminishes, and the displacement of aggregates is more sufficient, making it easy to achieve density of SMA-13 mixture [26]. Accordingly, the aggregate breakage of the VCM specimen is mainly concentrated in the upper part of the specimen near the top surface, the breakage of the other parts is less, and the aggregate breakage is closer to the breakage of the pavement core sample.

## 7 Conclusions

In this study, the performance of SMA-13 mixtures that were compacted using MCM, GCM, and VCM was compared. Their physical properties, mechanical properties, and gradation changes under different vibration compaction times, gyratory compaction numbers, and Marshall double-compaction numbers were discussed. The compaction method for SMA-13 mixture design was recommended under the principle of optimum properties. The main findings acquired from the above work were summarized as follows:

- The influence of vibration compaction time, gyratory compaction number, and Marshall double-compaction number on the density of SMA-13 mixture was analyzed. The Marshall double-compaction number corresponding to the density specified in the heavy traffic compaction standard was 155, which was called heavy MCM. The vibration compaction times corresponding to the density of MCM75 and MCM155 were 10 s and 65 s, respectively. The gyratory compaction numbers corresponding to the density of MCM50, MCM75, and MCM155 were 50, 65, and 130, respectively.
- Asphalt aggregate ratio and compaction work directly affect the volumetric properties (VV, VFA, and VMA) of asphalt mixture specimens while the raw material and mineral aggregate gradation were fixed. Under the same compaction work and the optimum asphalt aggregate ratio, the mechanical properties of GCM50 were 8% higher than those of MCM50; the mechanical properties of GCM65 and VCM10 were 10% and 13% higher than those of MCM75, respectively; the mechanical properties of GCM130 and VCM65 were 8% and 12% higher than those of MCM155, respectively.
- Under the same compaction method and the optimum asphalt aggregate ratio, if the compactness of the SMA-13 mixture increased by 1%, the Marshall stability, compressive strength, shear strength and splitting strength were improved on average by 8.1%– 11.3%, 6.5%– 8.0%, 14%– 18.0%, and 7.6%– 13.7%, respectively.
- The gradations of SMA-13 mixture were changed compared with the original gradation, which showed that the aggregates of the mixture were crushed during the compaction progress. The aggregate gradation of the SMA-13 mixture before and after compacted using VCM and GCM changed minimally compared with that of the SMA-13 mixture compacted using MCM. Compared with the breakage of the MCM specimen, the breakage of larger than 4.75 mm size aggregates of the GCM specimen was reduced by 1.9%, and that of the VGM specimen is reduced by 2.8% in the same compaction work.
- Through comparing the volume parameters, mechanical properties, and gradation changes of SMA-13 mixtures prepared under different compaction methods, the compaction methods of VCM65 and GCM130 were recommended for SMA-13 mixture design. They provide a reference for SMA-13 mixture design. The influence of these compaction methods on road performance, such as rutting-resistance and durability, will be evaluated in future studies.
